# DNA methylation clocks for clawed frogs reveal evolutionary conservation of epigenetic aging

**DOI:** 10.1007/s11357-023-00840-3

**Published:** 2023-06-04

**Authors:** Joseph A. Zoller, Eleftheria Parasyraki, Ake T. Lu, Amin Haghani, Christof Niehrs, Steve Horvath

**Affiliations:** 1 Department of Biostatistics, School of Public Health, University of California, Los Angeles, Los Angeles, CA USA; 2 Institute of Molecular Biology (IMB), Mainz, Germany; 3 Department of Human Genetics, David Geffen School of Medicine, University of California, Los Angeles, Los Angeles, CA USA; 4 Altos Labs, San Diego, CA USA; 5 German Cancer Research Center (DKFZ), Division of Molecular Embryology, DKFZ-ZMBH Alliance, Heidelberg, Germany

**Keywords:** Epigenetic clock, Methylation, Clawed frog, Biomarker of aging, Evolution

## Abstract

To address how conserved DNA methylation-based epigenetic aging is in diverse branches of the tree of life, we generated DNA methylation data from African clawed frogs (*Xenopus laevis*) and Western clawed frogs (*Xenopus tropicalis*) and built multiple epigenetic clocks. Dual species clocks were developed that apply to both humans and frogs (human-clawed frog clocks), supporting that epigenetic aging processes are evolutionary conserved outside mammals. Highly conserved positively age-related CpGs are located in neural-developmental genes such as *uncx*, *tfap2d* as well as *nr4a2* implicated in age-associated disease. We conclude that signatures of epigenetic aging are evolutionary conserved between frogs and mammals and that the associated genes relate to neural processes, altogether opening opportunities to employ *Xenopus* as a model organism to study aging.

**Supplementary Information:**

The online version contains supplementary material available at 10.1007/s11357-023-00840-3.

## Introduction

Stereotypical changes of DNA methylation (DNAm) of cytosine residues within CpG dinucleotides have emerged as one of the most reliable biomarkers of chronological age in mammals [1–7]. DNAm shows age-related signatures also in non-mammalian organisms, including chicken [8], frog [9], and the planktonic crustacean Daphnia [10]. This raises the important question how conserved are epigenetic aging signatures in non-mammalian vertebrates. Identification of epigenetic signatures will be highly informative in revealing molecular modules that enable subsequent functional exploration towards healthy aging both in amenable model organisms as well as in humans.

Amphibians are a widely used model system because of their experimental tractability and relatively closer evolutionary relationship with humans compared to alternatives such as fish or invertebrates. Among amphibian species, African clawed frog (*Xenopus laevis*) and the Western clawed frog (*Xenopus tropicalis*), are the molecularly best studied and their genomes have both been sequenced [11, 12]. *Xenopus* features epigenetic cytosine methylation via DNA methyltransferases *dnmt1* and *dnmt3a* and demethylation by *tet2* and *tet3* demethylases much like mammals [13–17].

While numerous studies concern the embryonic and larval development of *Xenopus*, the biology of adult aging has been little analyzed [18–22]. The main difficulty in studying aging in *Xenopus* is the long lifespan of *X. tropicalis* and *X. laevis*, estimated to be at least 16 and 30 years in captivity, respectively (own experience and [23]). In other amphibian species, studies on adult aging are mostly limited to lifespan analyses [24–28].

Hence, a reliable epigenetic clock biomarker may not only inform about conserved aging signatures, but also benefit the analysis of biological aging in this important model organism to facilitate DNAm-based assessments upon environmental exposures, for understanding of aging mechanisms, and for preclinical studies of anti-aging therapies.

Here, we generate *Xenopus* DNA methylation data across embryonic and larval stages as well as 6 different tissues from individuals ranging from embryos to 20-year-old adults. We were able to generate highly accurate epigenetic clocks for both *X. tropicalis* and *X. laevis*. Select CpGs in epigenetic aging signatures are shared between frogs and humans. The availability of *Xenopus* epigenetic clocks as an age biomarker overcomes the limits imposed by the decade lifetimes and may allow studying aging processes of tadpoles and juveniles in this well-established experimental model system.

## Results

### Two frog species

To ask, if we can establish epigenetic DNA methylation clocks for *Xenopus*, we first established a method to quantify genomic DNA methylation at sufficient sequencing depth. We used the mammalian methylation array platform that profiles individual CpGs in highly conserved stretches of DNA in mammals [29]. By design, the mammalian methylation array facilitates comparative studies across mammalian species (including humans) due to its very high sequencing depth (over thousand-fold) in highly conserved CpGs in mammals. This Infinium array measures up to 36 k CpGs per species that are well conserved across many mammalian species. It features a probe set that can tolerate specific cross-species mutations. The array was previously annotated in over 200 species and reports CpG island status and chromatin states.

Using this platform, we generated novel DNA methylation data and characterized DNAm from *X. laevis* (*n* = 35 samples) and *X. tropicalis* (*n* = 30 samples). We profiled six tissues (blood, brain, skin, liver, muscle, toe) as well as whole embryos, tadpoles, and juveniles, spanning a wide age range from 2-day- to 19-year-old whole animals and tissues, respectively (Suppl. Tables 1–3). Note that all samples used, with the exception of “toe” samples, represent multiple pooled individuals of the same age, thus yielding high-quality biological averages in each case. Despite evolutionary distance between frogs and mammals, 4635 CpGs out of 37,492 CpGs on the mammalian array map to one or both clawed frog genomes (1829 CpGs map to *X. laevis*, 4239 CpGs map to *X. tropicalis*) according to genome assemblies XenTro9.1.102 and XenLae10.1 from ENSEMBL.

**Fig. 1:**
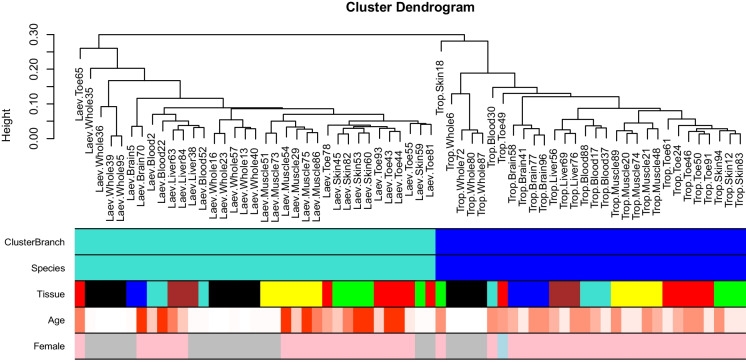
Unsupervised hierarchical clustering of *Xenopus* tissues. The clustering height (*y*-axis) can be interpreted as distance based on pairwise correlation coefficients. Color bands underneath indicate clustering branch (corresponding to a height cut-off of 0.29 on the *y*-axis), frog species (blue, tropicalis; turquoise, laevis), tissue type (see the labels), age (red corresponds to old age), and sex (pink, female; blue, male; grey, unknown). Sex was unknown for samples where the animals were too young to determine their sex

Unsupervised hierarchical clustering of the frog tissue samples (Fig. 1) shows that the samples fall into 2 distinct branches/clusters that correspond to species. Within species, some samples cluster by tissue type: e.g., muscle (yellow color), brain (blue color), and liver (brown) samples cluster together (third color band in Fig. 1). By contrast, skin and toe samples (red and green) seem to cluster together.

### Epigenetic clocks

Having established a method with high sequencing depth, we next asked if it is possible to construct epigenetic clocks for clawed frogs only and dual-species clocks for both human and clawed frogs. For the construction of the dual-species human-clawed frog clock, we used the DNAm data previously generated with the HorvathMammalMethylChip40 in 1366 human samples representing 20 tissues from individuals 0 to 101 years old [30]. Our *Xenopus* clocks can be distinguished along three dimensions: species, age range, and measure of age. We used a combined set of all samples to train pan-tissue clocks, for chronological age (pan-clock) and relative age (relative pan-clock), suited for age predictions across different tissue types included in the clock construction. The two species underlying our *Xenopus* clocks have markedly different maximum lifespans (30.3 for *laevis* and 16 for *tropicalis*) and average ages of sexual maturity (1 year for *laevis* and 0.375 for *tropicalis*). When building our *Xenopus* clocks, we addressed this fact in two ways. First, in our pan-clock, we used a log-linear transformation of age that effectively normalizes ages with respect to age at sexual maturity (Supplementary Methods). Second, in our relative pan-clock, we instead estimate relative age (chronological age divided by maximum lifespan), which normalizes ages with respect to maximum lifespan.

We also created clocks tailor-made for a specific species, which were trained based on all samples from the *laevis*-clock and the *tropicalis*-clock. It is important to note that, by construction, these clocks are designed to apply to a single species (*X. laevis* or *X. tropicalis*, respectively), and thus are not expected to validate when applied to any other *Xenopus* species. We also created a pan-tissue clock for young organisms (young-clock) trained on samples coming from animals no older than 2 years of age.

While the pan-tissue *Xenopus* clocks apply only to clawed frogs, we also created dual-species clocks, referred to as human-clawed frog clocks, for estimates of chronological age and relative age. Relative age is the ratio of chronological age to maximum lifespan (i.e., the maximum age of death observed in the species). Thus, relative age takes on values between 0 and 1. The maximum lifespan observed for *X. laevis* and *X. tropicalis* was 30.3 and 16 years, respectively, and the maximum lifespan observed for humans was 122.5 years. The “relative age” clock allows for alignment and biologically meaningful comparison between species with different lifespan (clawed frogs and humans), which is not afforded by mere measurement of chronological age. The “chronological age” human-clawed frog clock also accounts for the significant differences in lifespans; it uses a transformation of age within the formulation of the regression model, designed to vary the “speed” of the clock depending on the age of sexual maturity of the species (Supplementary Information).

**Fig. 2:**
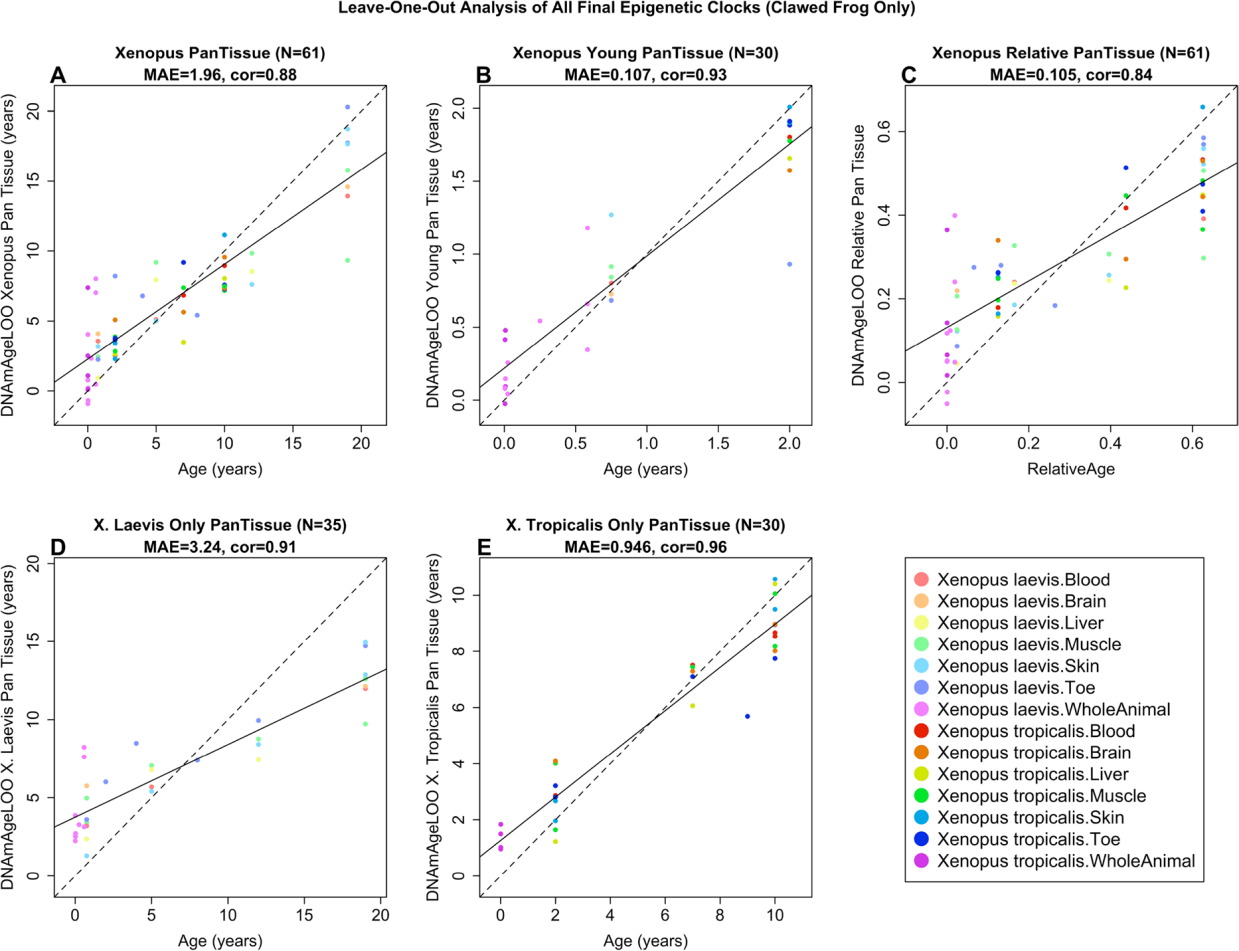
Cross-validation study of epigenetic clocks for *Xenopus*. Leave-one-sample-out estimate of DNA methylation age (*y*-axis, in units of years) versus chronological age (units of years) for **A** all *Xenopus* tissues (both species), **B** all tissues from young *Xenopus* (age < 2 years), **D** all tissues from *X. laevis*, **E** all tissues from *X. tropicalis*. **C** Leave-one-sample-out estimate of DNA methylation relative age (*y*-axis, values range from 0 to 1) versus relative age for all *Xenopus* tissues (both species). Relative age was determined by dividing chronological age (measured in years) by the maximum lifespan (also expressed in years). All clocks are pan tissue clocks, i.e., apply to all considered tissues Each panel reports the sample size (in parenthesis), correlation coefficient, median absolute error (MAE)

To arrive at unbiased estimates of the epigenetic clocks, we used leave-one-out (LOO) cross-validation of the training data. The cross-validation study reports unbiased estimates of the age correlation R (defined as Pearson’s correlation between the age estimate (DNAm age) and chronological age) as well as the median absolute error (MAE) measuring the deviation between the predicted and observed age (for chronological age in years). As indicated by their names, the pan-clock is highly accurate in age estimation of the different tissue samples (*R* = 0.88 and median error 1.96 years, Fig. 2A), as so is the relative pan-clock (*R* = 0.84 and median error 0.105 units, Fig. 2C), and the young-clock is highly accurate in age estimation of the different tissue samples coming from young animals (*R* = 0.93 and median error 0.107 years, Fig. 2B). We also developed highly accurate clawed frog clocks for single species: *X. laevis* (*R* = 0.91 and median error 3.24 years, Fig. 2D) and *X. tropicalis* (*R* = 0.96 and median error 0.946 years, Fig. 2E).

**Fig. 3:**
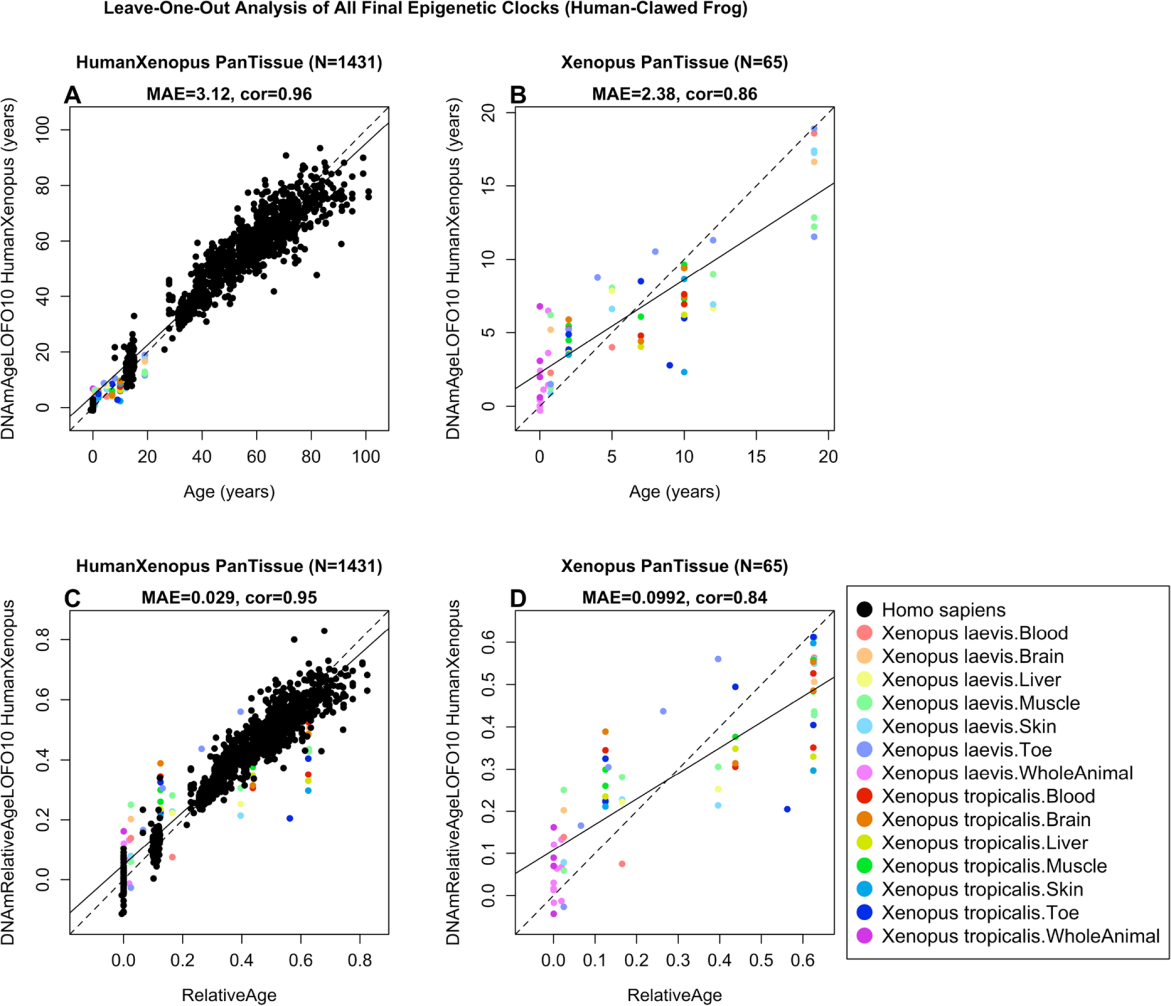
Cross-validation study of epigenetic clocks for *Xenopus* and humans. Ten-fold cross validation analysis of the human-clawed frog clocks for **A**, **B** chronological age and **C**, **D** relative age, respectively. **A**, **C** Human samples are colored in black and *Xenopus* samples are colored by species and tissue type, and analogous in **B**, **D** but restricted to *Xenopus* samples (colored by *Xenopus* tissue type). Relative age was determined by dividing chronological age (measured in years) by the maximum lifespan (also expressed in years). The relative age clock allows for alignment and biologically meaningful comparison between species with different lifespan (clawed frogs and humans). Each panel reports the sample size (in parenthesis), correlation coefficient, median absolute error (MAE)

We developed two dual-species clocks based on our clawed frog samples and previously characterized human tissues. While the first dual clock estimates chronological age, the second estimates relative age. The interest to create such dual-species clocks is (i) that they are expected to increase the likelihood that findings in one species will translate to the other, and (ii) to increase statistical significance. The human-clawed frog clocks are highly accurate, for both chronological age (*R* = 0.96 for the human and clawed frog samples and *R* = 0.86 for the clawed frog samples, Fig. 3A, B) and relative age (*R* = 0.95 for the human and clawed frog samples and *R* = 0.84 for the clawed frog samples, Fig. 3C, D).

### Epigenome-wide association study of age

**Fig. 4:**
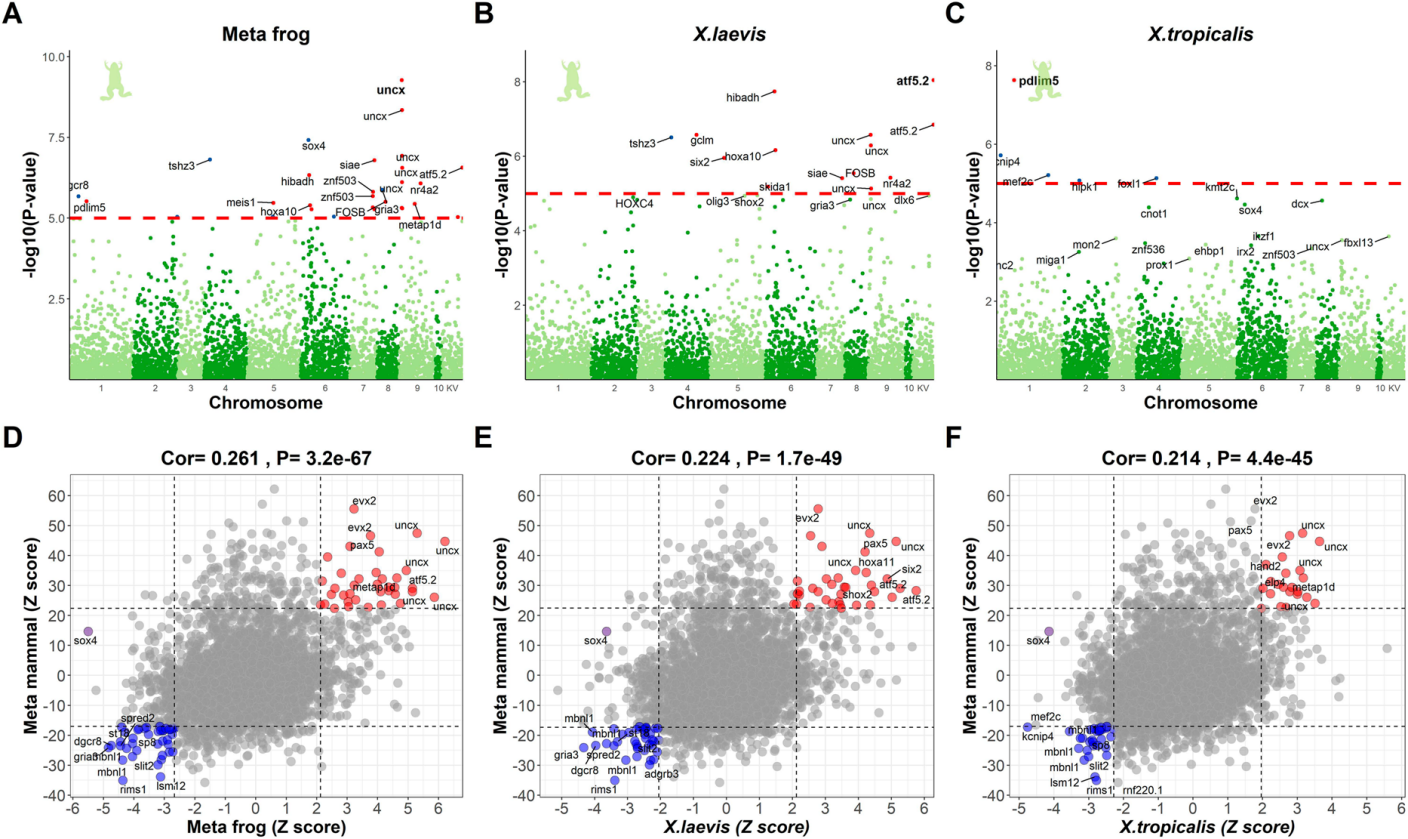
EWAS of age in *Xenopus*. The top panels display Manhattan plots for EWAS of age **A** Stouffer’s method meta-analysis that combines **B** *X. laevis*, and **C** *X. tropicalis* across all tissue types. The red dash lines indicate suggestive levels of significance at *P* < 1.0E-05. CpGs are colored in red or blue for positive or negative age correlations, respectively. The *y*-axis displays -log base 10 transformed *P*-value and the *x*-axis displays chromosome number based on the *X. tropicalis* genome (v9.1.102). Chromosome KV denotes the alias names for the CpGs with unspecified chromosomes. **D**–**F** Display the scatter plots between frog EWAS of age and Eutherian EWAS of age, based on Z statistics. Each of the 4239 points in the scatter plot correspond to a CpG that is present on our mammalian array and maps to the *X. tropicalis* genome. The title presents the Pearson correlation and its *P*-value between the two EWAS Z statistics. The CpG cg17865363 exhibiting highly significant *P*-value in frog EWAS is annotated by its nearby gene *sox4* and marked in purple. Labels are provided for the top 10 hypermethylated/hypomethylated CpGs according to the product of Z scores in *x*- and *y*-axis

We conducted an epigenome-wide association study (EWAS) to identify genes nearby methylated CpGs associated with aging. We performed three separate epigenome-wide association studies (EWAS) of age, which involved (i) *X. laevis* only (*n* = 35), (ii) *X. tropicalis* only (*n* = 30), and (iii) Stouffer meta-analysis combining the former two EWAS results (Fig. 4A–C; Suppl. Table 4). Due to the low sample size, we ignored tissue type in this analysis. Each EWAS correlated chronological age with individual CpG methylation levels. The analysis was restricted to 4239 CpGs that mapped to the genome of *X. tropicalis* (XenTro9.1.102).

At a genome-wide significance level of *P* < 1.0 × 10^−7^, two CpGs were significant in the *X. laevis* EWAS (Fig. 4B; Suppl. Table 4). One CpG cg10071691 (*P* = 9.1 × 10^−9^) is located near *atf5.2* on an unspecified chromosome (alias name: KV460357.1). The other one, cg11266179 (*P* = 1.8 × 10^−8^), is located near *hibadh*. In *X. tropicalis*, only one CpG (*P* = 2.3 × 10^−8^) near *pdlim5* reached genome-wide significance (Fig. 4C; Suppl. Table 4).

To combine the EWAS results of both *Xenopus* species, we used Stouffer meta-analysis method (equal weights) resulting in a Z statistic that follows a standard normal distribution under the null hypothesis of zero correlation with age. Comparing the frog EWAS results with those from the Mammalian Methylation Consortium reveals that CpGs in the *UNCX* gene are also highly correlated with chronological age in many mammalian tissues as shown in our pan-mammalian aging studies [31]. The EWAS of age results in frogs correlated positively (Pearson’s correlation *r* = 0.26) with the EWAS of age results in eutherian mammalian species (Fig. 4D; Suppl. Table 4) revealing an age-related gain of methylation in genes that play a role notably in neural development (e.g., *uncx*, *sox4*, *pax5*, and *evx2*). Similar correlation with the EWAS results from mammals could be observed when focusing the analysis on EWAS findings from a single frog species ($r$ > 0.2, Fig. 4E, F).

In the following, we refer to CpGs whose methylation increases and decreases with age, as “positive” and “negative” CpGs, respectively. *Uncx* was the top gene with positive CpG methylation-age correlation. Of the 4239 CpGs, 10 significantly age-related CpGs were located near *uncx*, mostly in the promoter (Fig. 4A, Suppl. Table 4). Human *UNCX* encodes a homeobox transcription factor whose mouse homolog is implicated in the development of the axial skeleton and in neural progenitor regulation in the olfactory epithelium [32, 33] and for which increased DNAm (cg04816311) is associated with target organ damage in older African Americans [34]. Notably, mutations in the *uncx* orthologue in *C.* *elegans*, *unc4*, extend male lifespan [35]. A positive age-related CpG was found near *hibadh*, where CpG methylation gain of human *HIBADH* (cg01065605) positively correlates with mortality in the InCHIANTI cohort [36]. For *nr4a2*, downregulation of the respective rat gene was reported in the hippocampus of aged animals [37].

**Fig. 5:**
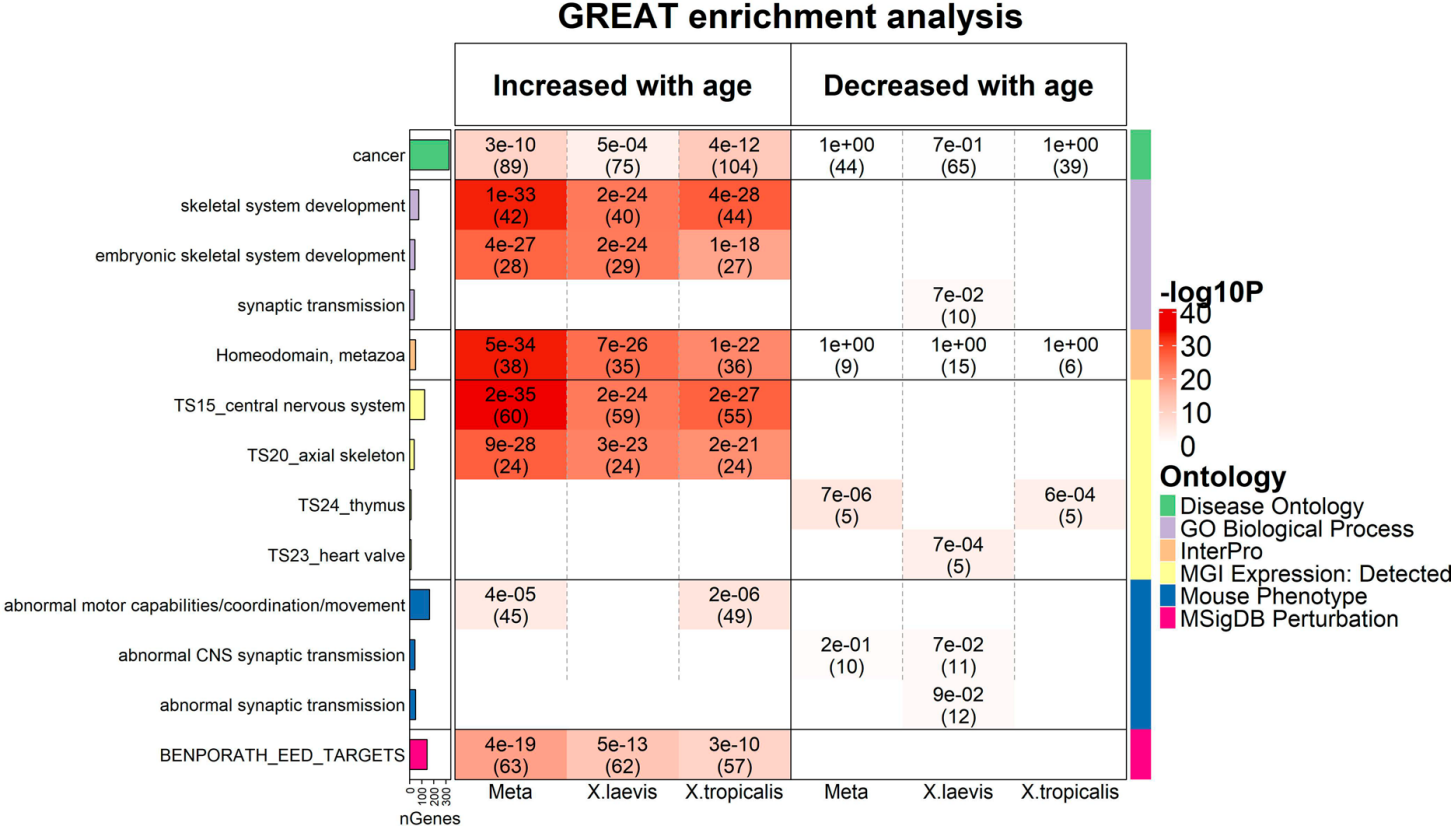
Genomic region-based GREAT functional enrichment analysis. GREAT functional enrichment analysis was based on the top 500 CpGs that increased or decreased with age from EWAS in (1) meta-analysis, (2) *X. laevis*, and (3) *X. tropicalis*, respectively (Suppl. Table 4). The background was based on the genomic regions of the 4239 mammalian CpGs and the assembly in hg19. The *y*-axis lists the name of a functional gene set/biological pathway, sorted by ontology and the most significant hypergeometric *P*-value within each ontology. The bar plots in the first column report the total number of genes at each studied gene set adjusted based on our background. The left and right panels of the *x*-axis list the enrichment results based on the top 500 CpGs with positive and negative age correlation. We list unadjusted hypergeometric *P*-value (number of overlap genes) at each cell, provided *P* < 0.1. The heatmap color codes -log10 (*P*-value). Abbreviations: BENPORATH_EED_TARGETS denotes “EED targets: genes identified by ChIP on chip as targets of the Polycomb protein EED (GeneID = 8726) in human embryonic stem cells.

The top-ranking negative age-related CpG is located near *sox4* (*SRY-box transcription factor 4*), which regulates retinal precursor development in *Xenopus* [38]. DNAm or expression of *SOX4* in mice and humans are associated with aging or age-related disease, notably cancer [39, 40]. A negative age-related CpG is located near *tshz3* (*Teashirt Zinc Finger Homeobox 3*), rare protein-altering variants of which are highly enriched in a cohort of supercentenarians [41]. It is noteworthy that negative age-related CpG associated gene orthologs tend to be involved in synaptic transmission, including *tshz3*, *dgcr8*, *spred2*, *gria3*, *gria4*, *kcnip4,* and *rims1* [42–47]*.* Indeed, the term “synaptic transmission” was retrieved in GREAT functional enrichment analysis among the genes that lose methylation with age (Fig. 5). Once again, these genes are also associated with negative age-related CpGs in mammals [31, 48, 49].

### Functional enrichment of EWAS of age

To annotate the biological functions of the age-related CpGs in frogs, we performed genomic region GREAT functional enrichment analysis [50]. The functional annotations were based on the top 500 positive and the top 500 negative age-related CpGs from each of the 3 EWAS studies. The background underlying the GREAT analysis was based on the genomic regions of the 4239 CpGs in Hg19 assembly. Choosing the 4239 CpGs has background set ensured that our enrichment analyses were not biased by the content/design of the mammalian array. We used the human annotation Hg19 assembly as coordinate system since we were interested in comparing the results in frogs to those from humans but we acknowledge that this approach has limitations.

The positive age-related CpGs were enriched in several gene sets involved in developmental processes, notably embryonic skeletal system development in GO term (GREAT *P*-value < 1.0 × 10^−33^), target sites of Polycomb repressive complex 2 (PRC2) such the subunit EED in the MSigDB database (“BENPORATH_EDD_TARGETS,” *P*-value < 1.0 × 10^−19^, Fig. 5, Suppl. Table 5). Similarly, we find enrichments for gene sets that play a role in the development of mice including axial skeleton and skeleton or rib morphology (Fig. 5). Moreover, “homeodomain” and genes expressed in mouse central nervous system were highly enriched terms (GREAT *P*-value 5 × 10^−34^ and 2 × 10^−35^, respectively). We also submitted all 4239 CpGs to GREAT analysis, using the entire mammalian array as background. We did not find strong enrichments associated with PRC2 binding sets (*P* > 0.01) or “homeodomain” (*P* > 0.001), indicating that the “PRC2” and “homeodomain” enrichments (Fig. 5) are not confounded by the background.

**Fig. 6:**
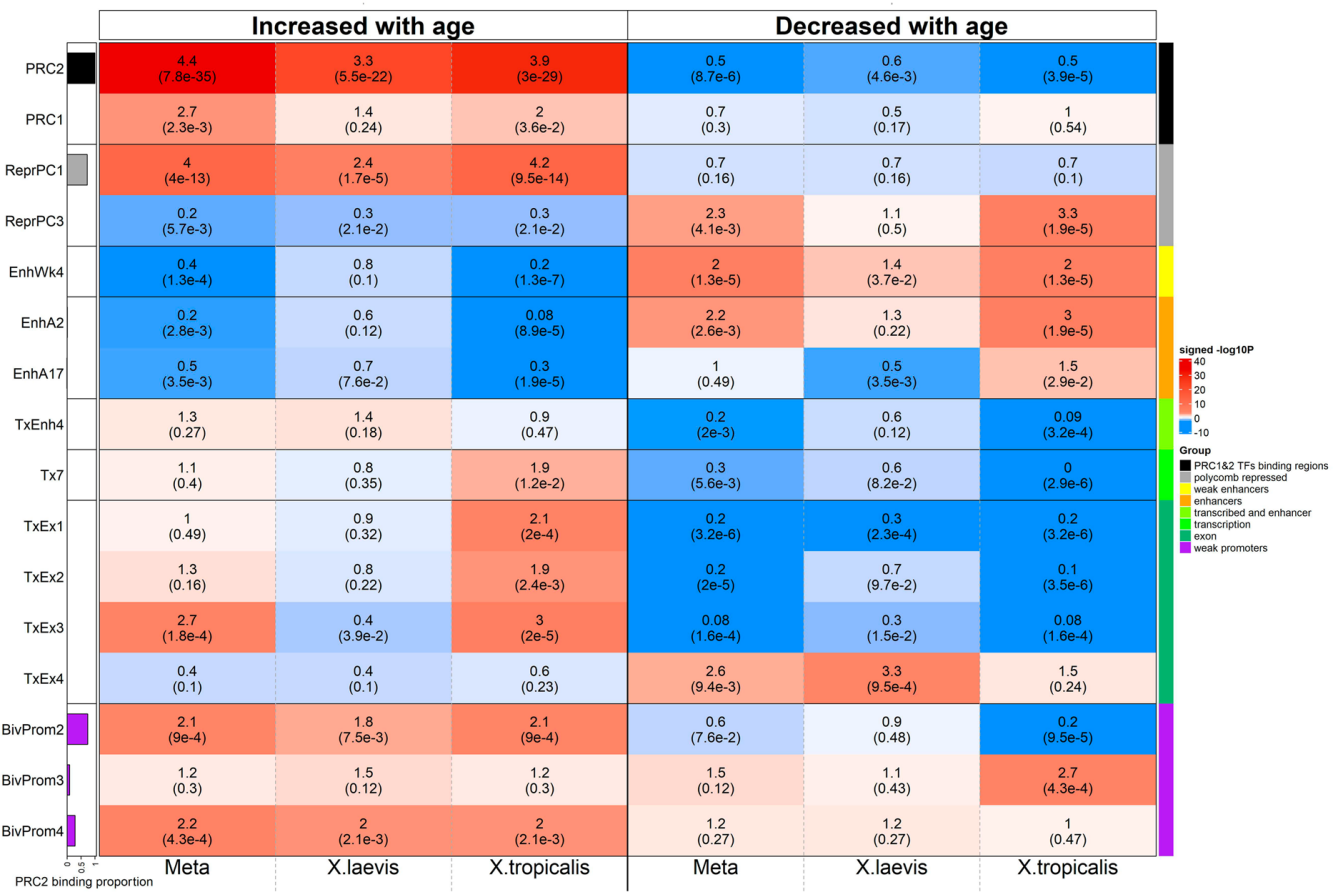
Chromatin state analysis of age-related CpGs. The heatmap color-codes the hypergeometric overlap analysis between age-related CpGs (columns) and two groupings of CpGs (a) binding by Polycomb repressive complex 1 and 2 (PRC1, PRC2) defined based on ChipSeq datasets in ENCODE [64] and (b) universal chromatin states analysis [61], see the first two rows. The background is based on the 4239 mammalian CpGs that can map to the *X. tropicalis* genome (v9.1.102). The first column shows a bar plot that reports the proportion of CpGs bound by PRC2 that ranges from zero (RPC1) to one (PRC2). For each row (chromatin state or PRC annotation), the table reports odds ratios (OR) from hypergeometric test results for the top 500 CpGs that increased/decreased with age from meta-EWAS, *X. laevis* EWAS and *X. tropicalis* EWAS, respectively. The heatmap color gradient is based on -log10 (unadjusted hypergeometric *P*-value) multiplied by the sign of OR greater than one. Red colors denote OR greater than one in contrast with blue colors for OR less than one. Legend lists states based on their group category and PRC group. The *y*-axis lists chromatin states and PRC2 target sites. The left/right panel lists the results based on the top 500 CpGs with positive/negative age correlation. We display 16 universal chromatin states that show significant enrichment/depletion at *P* < 0.001 in any of the EWAS

Fewer significant enrichments can be observed for negatively age-related CpGs. The identified gene sets include GO terms such as regulation of synaptic transmission, RNA splicing, and genes expressed in the thymus or heart valve according to MGI expression, regulation of RNA splicing under GO term (Figs. 5 and 6).

### Human chromatin state annotation

Given that key gene regulatory mechanisms and also chromatin states are evolutionary conserved among vertebrates [51, 52], we were encouraged to use the vast *human* chromatin state data available and map them to the EWAS of age results in frogs. Indeed, the findings in frogs turned out to be consistent with those from the EWAS of age by the Mammalian Methylation Consortium [31] as detailed in the following. We used universal human chromatin states based on 1032 experiments that mapped 32 types of chromatin marks in over 100 human cell and tissue types [53]. First, we used the hypergeometric test-based overlap analysis between chromatin states and the top 500 *positively* age-related CpGs. Again, the analysis properly adjusted for the background set of CpGs that map to the frog genome and the array platform.

For the positive age-related CpGs, we observed significant overlap with bivalent regulatory regions (specifically bivalent promoter 2 and bivalent promoter 4) and a Polycomb repressed state (ReprPC1). These three states contain a high proportion of CpGs at target sites of Polycomb repressive complex 2 (PRC2).

The frog meta-analysis EWAS of age exhibits significant overlap with BivProm2 state with an odds ratio greater than 2 (hypergeometric *P* = 9.0 × 10^−4^, Fig. 6 and Suppl. Table 6). In contrast, the negatively age-related CpGs (top 500) are enriched in select weak enhancer state EnhWk4, enhancer state EnhA2 and transcribed exon state TxEx4.

### Overlap with PRC2 target sites

Since a hallmark of positive age-related CpGs in mammals is their association with regions that are targeted by PRC2 [2, 54, 55], we further examined the overlap between the age-related CpGs and target sites of both PRC1 and PRC2. Toward this end, we used PRC binding annotations from human cells, as we found that the available *Xenopus* PRC2 binding data sets [56] did not provide enough sequencing coverage at CpGs on the mammalian array: only 50 CpGs on the mammalian methylation array map to H3K27me3 sites in *Xenopus*. Similarly, only 2 CpGs map to EZH2 sites in *Xenopus*. Using human annotations, we defined PRC sites based on the binding of at least two transcription factor members of PRC1 (subunits: RING1, RNF2, BMI1) or of PRC2 (subunits: EED,SUZ12 and EZH2) in 49 ChipSeq datasets available in ENCODE [57]. The top 500 CpGs with positive age correlation are enriched in PRC2 target sits based on the meta EWAS (hypergeometric *P*-value = 7.8 × 10^−35^ and odds ratio (OR) = 4.4), *X*. *laevis* EWAS (OR = 3.3 and *P* = 5.5 × 10^−22^) and *X. tropicalis* EWAS (OR = 3.9 and *P* = 3.0 × 10^−29^, Fig. 6 and Suppl. Table 6). By contrast, the overlap with PRC1 target sites is far less significant (Fig. 6). The results suggest that the association of aging-related methylated CpGs with PRC2 target genes is evolutionary conserved between mammals and amphibians.

## Discussion

The key findings of this study are, first, that it is possible to construct an epigenetic clock for frogs and, second, the evolutionary conservation of epigenetic aging signatures between frogs and humans. This evolutionary conservation relates to (i) our ability to construct two epigenetic clocks that apply to *Xenopus* and humans, (ii) that age-related CpGs are located near genes also found in mammalian clocks and that are implicated in age-associated disease, (iii) that positive age-related CpGs are associated with PRC2 target sides, a hallmark also observed in mammals. Overall, these epigenetic clocks provide the first accurate age biomarker in *Xenopus* and open the possibility to study aging processes in tadpoles and juveniles in this well-established experimental model system.

A striking finding of this study is the construction of two epigenetic clocks that apply to *Xenopus* and humans. The two clocks have different interpretations: the first clock measures chronological age in both species. The second clock measures relative age (age divided by maximum lifespan). Relative age may be a biologically more meaningful measure since it adjusts for the strong difference in lifespan. Each of these dual-species clocks estimates age based on a single mathematical formula derived from a multivariate regression model focusing on highly conserved CpGs. The fact that we could successfully construct human-clawed frog clocks is due to both biological and technical reasons. A biological reason is the high conservation of (positively) age-related changes in PRC2 target sites, as can be seen from our EWAS of age. A technical reason is the large sequencing depth at highly conserved CpGs that were profiled on the mammalian methylation array platform [29]. Our dual species human-clawed frog clocks, for absolute and relative age, increase the chance that findings in frogs translate to humans and vice versa. The bias due to differences in maximum lifespan is mitigated by the generation of the human-clawed frog clocks for *relative* age, which embeds the estimated age in the context of the maximal lifespan recorded for the relevant species. The high accuracy of these clocks demonstrates that one can build epigenetic clocks for two species based on a single mathematical formula. Treatments that alter the epigenetic age in *Xenopus* are therefore likely to exert similar effects in humans.

We also present *Xenopus* clocks that were trained in *X. laevis* and *X. tropicalis*. We generated DNA methylation data from six tissue types and from whole animals in these two species. Using these DNAm data, we trained and validated highly accurate age estimators (epigenetic clocks) that apply to the developmental life course (from birth to mid-life), and identified genes associated with the aging process in the clawed frog. These data allowed us to construct a highly accurate pan-tissue age estimator (pan-clock) based on six clawed frog tissue types (blood, brain, liver, muscle, skin, toe) and whole animal, and clocks developed based on individual *Xenopus* species, as well as a clock developed based on all tissues from embryos and juveniles (young-clock). Given that all four pure *Xenopus* clocks can estimate age in six different tissues, we anticipate that they apply to additional tissues as well. However, we cannot rule out that these clocks could fail in some highly specialized cell types. We expect that the *Xenopus* clocks apply to other clawed frog species as well in the sense that they will lead to high age correlations in tissue samples collected from both young and old frogs. However, epigenetic age predictions can differ from the true chronological age by a constant offset/bias in new tissue types or new frog species due to biological and technical reasons including differences in probe sequence conservation or storage conditions. The offset/bias can be estimated from the data by including tissue samples from frogs of known ages.

A limitation of our study is that the mammalian array profiles only about 4239 CpGs out of millions of CpGs in the *X. tropicalis* genome. While the large sequencing depth at highly conserved CpGs is ideal for building human-frog clocks, the low number of cytosines renders the interpretation of EWAS analyses tentative. The low CpG number will also be limiting for studies that aim to characterize regulatory relationships between methylation and transcriptomic changes.

Altogether, these epigenetic clocks reveal several salient features with regard to the biology of aging. First, the *Xenopus* pan-tissue clock reaffirms the conclusion drawn from the human pan-tissue clock, which is that aging might be a systemic biological process that affects the whole body. Second, the ability to combine these two pan-tissue clocks into a single human-clawed frog pan-tissue clock, species whose lineages diverged some 350 million years ago (according to timetree.org) [58], attests to the high conservation of the aging process. This conclusion is corroborated by our EWAS analysis.

Previous studies in humans showed that a hallmark of age-related CpGs is their association with target sites of PRC2, which gain methylation with age [2, 54, 55] and this feature is fully recapitulated in *Xenopus*. The physiological significance of this association is an important open question. PRC2 plays a prominent role during embryonic development [59] and consequently, many aging-clock-associated genes relate to developmental processes. Given its evolutionary conservation from frogs to human, the methylation status of PRC2 targets supports some critical causal relationship to systemic aging. Since the association with PRC2 with aging stems from analyses of adult, postmitotic cells and of different tissue origin rather than from embryonic cells, is tempting to speculate that the adult methylation status will get important input during embryonic development, the very phase when PRC2 target gene expression is prominent. Indeed, according to *Xenopus* epigenetic clocks, epigenetic aging proceeds already during embryonic and larval development, long before metamorphosis, which only begins weeks after fertilization. Consistent with the idea of “embryonic aging,” *Xenopus* genes associated with positive age-related CpGs encode many developmental regulators. In particular, it is noteworthy that genes associated with both positive and negative age-related CpGs relate to neural processes, although in somewhat opposite direction: while DNAm increase is linked to neural developmental genes, DNAm decrease links to synaptic transmission, roughly corresponding to processes of immature vs. mature neuronal cells, respectively. Altogether, this leads to the counter-intuitive suggestion that studying *Xenopus* neural development may yield new insights into biological aging. The availability of epigenetic clocks as quantitative, accurate age biomarker overcomes the limitations set by the decade lifetimes of clawed frogs and render this endeavor feasible.

## Methods

### Ethics statement

*Xenopus* experiments were approved by the state review board of Rheinland Pfalz, Germany, (Landesuntersuchungsamt, reference number 23177–07/ A17-5-002 HP) and performed according to federal and institutional guidelines.

### Study subjects

Adult frogs were obtained from three sources: NASCO Education, European *Xenopus* Resource Centre (EXRC) and National *Xenopus* Resource (NXR). Embryos, tadpoles, and juvenile animals were prepared at the Institute of Molecular Biology (IMB) facility in Mainz by in vitro fertilization as described in [60]. *X. laevis* and *X. tropicalis* embryos were cultivated in 0.1 × Barth’s solution and grown between 18 and 23 °C, respectively. Adult *X. laevis* were kept at 18 °C and adult *X. tropicalis* at 25 °C with a light/dark cycle of 12 h/12 h.

### Xenopus tissue samples

For this study, we analyzed a total of *n* = 65 samples, representing the development of *Xenopus*, from neurula to mid-life adult stages. We analyzed *n* = 35 tissues from *X. laevis* and *n* = 30 from *X. tropicalis*, coming from the same set of tissue types. In *X. laevis*, we analyzed samples from peripheral blood (*n* = 3), brain (*n* = 2), liver (*n* = 3), hind limb thigh muscle (*n* = 6), skin (*n* = 5), toes (*n* = 7), and whole animal (*n* = 9). In *X. tropicalis*, we analyzed samples from peripheral blood (*n* = 4), brain (*n* = 4), liver (*n* = 3), hind limb thigh muscle (*n* = 5), skin (*n* = 4), toes (*n* = 6), and whole animal (*n* = 4). Samples from whole animal were from whole embryos and juveniles NF stage 18, 28, 47, 55, 58, and 66 in *X. laevis* and stage 18 and 28 in *X. tropicalis*.

Animals were anesthetized in 0.15% MS-222 (Sigma-Adrich, A5040) and sacrificed by transection between the brainstem and the spinal cord. After harvesting, the tissues were snap frozen in liquid nitrogen and grinded to powder using a mortar and a pestle. For genomic DNA extraction, 25 mg tissue were mixed with 20 μl 20 mg/ml Proteinase K (Qiagen, 19131) and 180 μl buffer ATL (Qiagen, 19076) and incubated for 1 h at 56 °C, 1000 rpm at an Eppendorf Thermomixer Comfort. For genomic DNA extraction from blood, we used 10 μl peripheral blood. DNA extraction was performed using the DNeasy Blood & Tissue kit (Qiagen, 69504). DNA was eluted in 100 μl buffer AE (10 mM Tris–Cl, 0.5 mM EDTA; pH 9.0). Samples with DNA concentration below 50 ng/μl were concentrated by ethanol precipitation. The DNA pellet was washed with 70% ethanol and resuspended in 50 μl AE buffer.

### Human tissue samples

To build the human-clawed frog clock, we analyzed previously generated methylation data from *n* = 1366 human tissue samples (adipose, blood, bone marrow, cerebellum, cortex, dermis, epidermis, embryonic cells, fibroblasts, heart, keratinocytes, kidney, liver, lung, lymph node, muscle, pituitary, placenta, skin, spleen) from individuals whose ages ranged from 0 to 101 years [30]. The tissue samples came from three sources. Tissue and organ samples were from the National NeuroAIDS Tissue Consortium [61]. Blood samples were from the Cape Town Adolescent Antiretroviral Cohort study [62]. Skin and other primary cells were provided by Kenneth Raj [63]. Ethics approval (IRB#15-001454, IRB#16-000471, IRB#18-000315, IRB#16-002028).

### DNA methylation data

All DNA methylation data were generated using the mammalian Infinium array “HorvathMammalMethylChip40” [29]. By design, the mammalian methylation array facilitates epigenetic studies across mammalian species (including humans) due to its very high coverage (over thousand-fold) of highly conserved CpGs in mammals. A subset of cytosines on the mammalian array also applies to more distant species including amphibians [29].

The Infinium method is based on sodium bisulfite conversion of DNA and microarray-based genotyping of CpG sites using Infinium bead technology with single-base resolution. The advantage of the microarray platform is that it is user-friendly, it can be multiplexed, and it exhibits good agreement with other platforms’ DNA methylation measures. Specifically, the Infinium beads bear a 23-base oligo address to locate them on the BeadChip, and a 50-base probe. Probe sequences are complementary to specific 50 base regions of bisulfite-converted genomic DNA. The 3′ end of the probe harbors the methylated CpG site to be monitored. After the probe is hybridized to bisulfite-treated test DNA, a single-base extension adds a fluorescently labeled ddNTP to the 3′ CpG site. This lets the C to T change caused by bisulfite conversion to be “genotyped.” The fluorescent signal is then measured and processed.

Out of all CpGs on the mammalian array, 4635 CpGs map to one or both of the clawed frog genomes (1829 map to African clawed frog, 4239 map to Western clawed frog) according to genome assemblies XenTro9.1.102 and XenLae10.1 from ENSEMBL. Genome coordinate information can be downloaded from our GitHub page (https://github.com/shorvath/MammalianMethylationConsortium) and the supplementary information in [29]. The chip manifest file can be found at Gene Expression Omnibus (GEO) at NCBI as platform GPL28271. The SeSaMe normalization method was used to define beta values for each probe [64].

### Unsupervised hierarchical clustering

Following hierarchical clustering, low-quality outlier samples were excluded from further analysis. As dissimilarity, we used 1 minus the Pearson’s correlation coefficient across the 4635 CpGs that map to tropicalis and/or laevis. We used average linkage as an intergroup dissimilarity measure (Fig. 1).

### Penalized regression models

Details on the clocks (CpGs, genome coordinates) and R software code are provided in Suppl. Table 7. Penalized regression models were created with glmnet [65]. We investigated models produced by “elastic net” regression (alpha = 0.5). The optimal penalty parameters in all cases were determined automatically by using a 10-fold internal cross-validation (cv.glmnet) on the training set. By definition, the alpha value for the elastic net regression was set to 0.5 (midpoint between Ridge and Lasso type regression) and was not optimized for model performance.

We performed a cross-validation scheme for arriving at unbiased (or at least less biased) estimates of the accuracy of the different DNAm-based age estimators. For validation of the clocks, we used leave-one-out LOO cross-validation (LOOCV) in which one sample was left out of the regression, then predicted the age for the remaining samples and iterated this process over all samples.

A critical step is the transformation of chronological age (the dependent variable). While no transformation was used for the single-species pan-tissue clocks for *X. laevis* and *X. tropicalis*, respectively, we did use a log-linear transformation for the 2-species pan-tissue clocks for clawed frogs and for the dual-species clock of chronological age (Supplement).

It is important to make clear here that the set of CpGs used to train these clocks is not the same set of CpGs that map to one of both of the clawed frog genomes according to genome assemblies. Rather, the CpGs presented to the regression model were those that were considered detectable, based on mean methylation values (Supplement).

### Relative age estimation

To introduce biological meaning into age estimates of two clawed frog species and humans that all have a very different lifespan, as well as to overcome the inevitable skewing due to unequal distribution of data points from clawed frogs and humans across age range, relative age estimation was made using the formula: Relative age = Age/maxLifespan, where the maximum lifespan for the three species was chosen from the *anAge* database [23]. Maximum age of African clawed frogs and Western clawed frogs was 30.3 and 16 years, respectively, and the maximum age of humans was 122.5 years. The oldest frog *X. tropicalis* (16 years) was still alive in the lab of Christof Niehrs in July 2022.

### Epigenome wide association studies of age

EWAS was performed in each tissue and frog species separately with the R function “standardScreeningNumericTrait” in the “WGCNA” R package [66]. Next, the results were combined across tissues with Stouffer’s meta-analysis method and combined across species.

### GREAT functional enrichment analysis

We used the Genomic Regions Enrichment of Annotations Tool (GREAT) to analyze the age-related CpGs [50]. This software tool has not yet been adapted for frogs. Rather, we used the human hg19 genome assembly.

To avoid biases arising from the use of the mammalian array platform, we restricted the background according to the genomic regions covered by the 4635 probes that mapped to the *X. tropicalis* genome. GREAT calculates statistics by associating genomic regions with nearby genes and applying the gene annotations to the regions. Association is a two-step process. First, every gene is assigned a regulatory domain. Then, each genomic region is associated with all genes whose regulatory domain it overlaps. To define the gene regulatory domain, each gene is assigned a basal regulatory domain of a minimum distance upstream and downstream of the TSS (regardless of other nearby genes). We used the settings: Proximal: 5.0 kb upstream, 1.0 kb downstream, plus Distal: up to 50 kb). Gene set enrichment was done for gene ontology, molecular pathways, diseases, upstream regulators, and human and mouse phenotypes.

### Genome annotation

We aligned microarray probes to the reference genomes of Xenopus_tropicalis 9.1.102 and Xenopus_laevis 10.1 from ENSEMBL. The alignment was done using the QUASR package [67], with the assumption of bisulfite conversion treatment of the genomic DNA. Following the alignment, the CpGs were annotated based on the distance to the closest transcriptional start site using the ChIPseeker package [68] (Suppl. Table 4).

### Supplementary Information

Below is the link to the electronic supplementary material.

Supplementary file1 (DOCX 34 KB)

Supplementary file2 (XLSX 3367 KB)

## Data Availability

The data will be made publicly available on Gene Expression Omnibus as part of the data release from the Mammalian Methylation Consortium. The manifest file of the mammalian array and genome annotations of the CpGs can be found on Github (https://doi.org/10.5281/zenodo.7574747) https://github.com/shorvath/MammalianMethylationConsortium. The mammalian methylation array is broadly available to the research community from the non-profit Epigenetic Clock Development Foundation (https://clockfoundation.org/).
